# A Novel Aminothiazole KY-05009 with Potential to Inhibit Traf2- and Nck-Interacting Kinase (TNIK) Attenuates TGF-β1-Mediated Epithelial-to-Mesenchymal Transition in Human Lung Adenocarcinoma A549 Cells

**DOI:** 10.1371/journal.pone.0110180

**Published:** 2014-10-22

**Authors:** Jiyeon Kim, Seong-Hee Moon, Bum Tae Kim, Chong Hak Chae, Joo Yun Lee, Seong Hwan Kim

**Affiliations:** 1 Department of Biomedical Laboratory Science, School of Medicine, Eulji University, Jung-gu, Daejeon, Republic of Korea; 2 Laboratory of Translational Therapeutics, Korea Research Institute of Chemical Technology, Yuseong-gu, Daejeon, Republic of Korea; 3 Division of Drug Discovery Research, Korea Research Institute of Chemical Technology, Yuseong-gu, Daejeon, Republic of Korea; 4 Drug Discovery Platform Technology Team, Korea Research Institute of Chemical Technology, Yuseong-gu, Daejeon, Republic of Korea; University of Birmingham, United Kingdom

## Abstract

Transforming growth factor (TGF)-β triggers the epithelial-to-mesenchymal transition (EMT) of cancer cells via well-orchestrated crosstalk between Smad and non-Smad signaling pathways, including Wnt/β-catenin. Since EMT-induced motility and invasion play a critical role in cancer metastasis, EMT-related molecules are emerging as novel targets of anti-cancer therapies. Traf2- and Nck-interacting kinase (TNIK) has recently been considered as a first-in-class anti-cancer target molecule to regulate Wnt signaling pathway, but pharmacologic inhibition of its EMT activity has not yet been studied. Here, using 5-(4-methylbenzamido)-2-(phenylamino)thiazole-4-carboxamide (KY-05009) with TNIK-inhibitory activity, its efficacy to inhibit EMT in cancer cells was validated. The molecular docking/binding study revealed the binding of KY-05009 in the hinge region of TNIK, and the inhibitory activity of KY-05009 against TNIK was confirmed by an ATP competition assay (*K*_i_, 100 nM). In A549 cells, KY-05009 significantly and strongly inhibited the TGF-β-activated EMT through the attenuation of Smad and non-Smad signaling pathways, including the Wnt, NF-κB, FAK-Src-paxillin-related focal adhesion, and MAP kinases (ERK and JNK) signaling pathways. Continuing efforts to identify and validate potential therapeutic targets associated with EMT, such as TNIK, provide new and improved therapies for treating and/or preventing EMT-based disorders, such as cancer metastasis and fibrosis.

## Introduction

Epithelial-to-mesenchymal transition (EMT) is the complicated process of change that epithelial cells undergo to acquire the characteristics of mesenchymal cells during embryogenesis, development, wound healing, organ fibrosis, and cancer metastasis [1], [2]. As they undergo EMT, the polarized and closely packed epithelial cells become more motile and invasive, which are the main characteristics of spindle-shaped mesenchymal cells. In cancer cells, particularly, EMT-induced motility and invasion play critical roles in the process of metastasis. Since metastasis is the major cause of death in cancer patients, the signaling molecules involved in the process of EMT are emerging as important, new therapeutic targets for inhibition.

Transforming growth factor (TGF)-β is the major cytokine that triggers EMT during cancer progression and metastasis [3]–[6]. The binding complex of TGF-β with its transmembrane Ser/Thr receptors, TGF-β type I (TβR-I) and type II (TβR-II), transphosphorylates TβR-I, and subsequently phosphorylates the downstream molecules, Smad2 and Smad3. The phosphorylated Smad2/3 complex recruits Smad4, then this trimeric complex further translocates into the nucleus, where it binds to transcription factors, such as Snail/Slug and Twist, to activate TGF-β-responsive genes [7]–[12].

TGF-β-activated, non-Smad signaling pathways, including the Wnt/β-catenin signaling pathway, are also required for EMT induction in cancer cells [13]–[15]. The canonical Wnt signaling pathway is mediated by the DNA-binding, HMG box transcription factors, lymphoid enhancer-binding factor 1 and T cell-specific factor (LEF1/TCF), and their coactivator, β-catenin. Aberrant Wnt signaling is well known for its involvement in cancer progression and metastasis [16], [17]. The interdependence between Smad and Wnt/β-catenin signaling pathways has been reported in several studies; the Snail family promotes formation of a β-catenin-TCF4 transcription complex to regulate the expression of TGF-β3 [15]; and TGF-β-dependent activation of LEF1/TCF target genes requires both Smad and LEF1/TCF DNA-binding sites [18]. Additionally, the functional blockade of Smad4 leads to decreased β-catenin levels [19]. Taken together, these data suggest that TGF-β triggers EMT through the cross-talk between Smad and Wnt/β-catenin signaling pathways. Thus, we hypothesize that the molecules, such as kinases, which directly control both signaling pathways, could effectively regulate the process of EMT.

**Figure 1:**
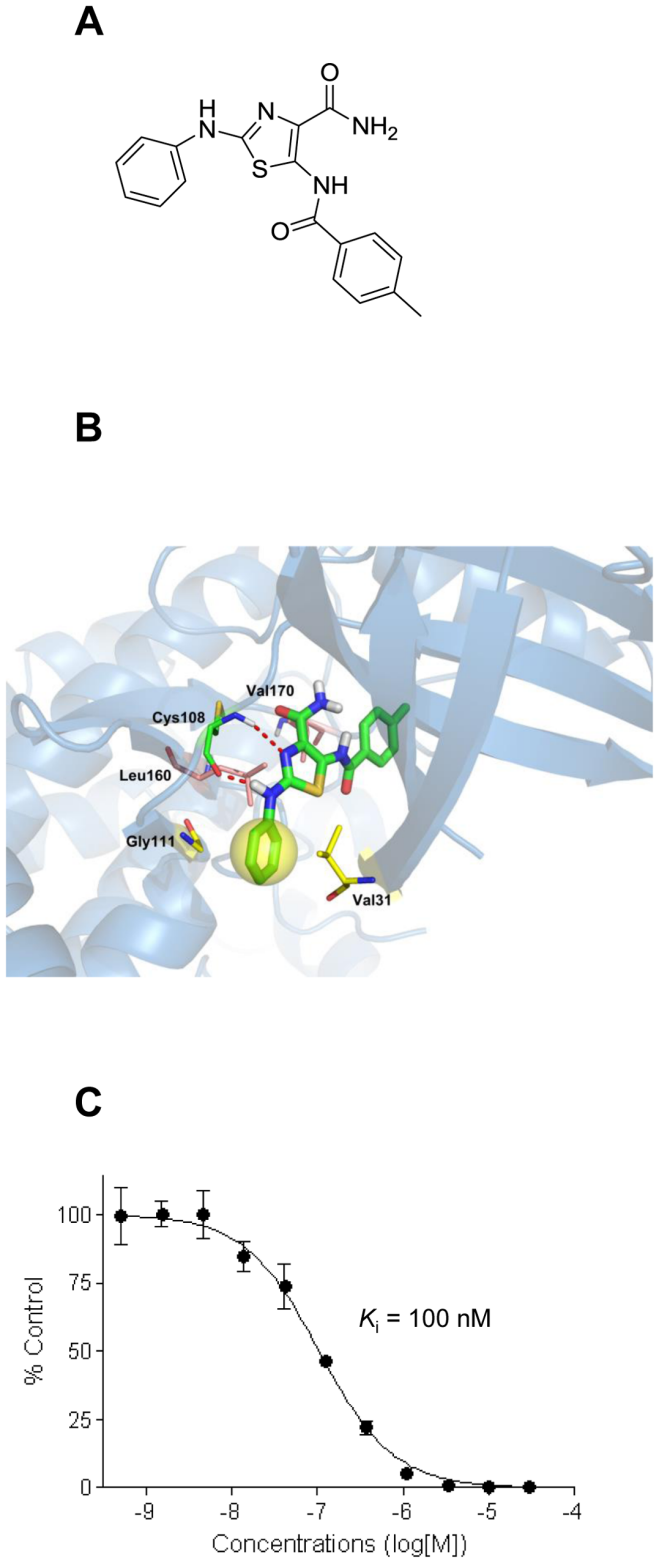
Binding mode and *K*_i_ of KY-05009 for TNIK. (A) Chemical structure of KY-05009. (B) Binding mode of KY-05009 for TNIK. KY-05009 has two H-bond interactions with Cys108 (red dotted lines) in the hinge region, and CH/π interactions with Val31, Gly111, and Leu160. The yellow ball represents the CH/π interactions among Val31, ligand, and Gly111. (C) The binding constant, *K*_i_, of KY-05009 for TNIK was determined using an ATP competition assay.

Recent reports have proposed the Traf2- and Nck-interacting kinase (TNIK) as a first-in-class anti-cancer target molecule [20]. A functional study revealed that its kinase activity is essential for the maintenance of colorectal cancer growth [21]. Through the interaction with TCF4 and β-catenin, TNIK phosphorylates TCF4 at serine 154, and mediates the activation of Wnt target genes that are involved in cancer cell growth [20], [21]. Additionally, TNIK has been identified as one of the kinases responsible for α-helix 1 phosphorylation of Smad, thus, causing its inhibition [22]. Although only a few biological activities of TNIK are involved in controlling the Wnt and Smad signaling pathways, we believe that it might be valuable in the early stage of drug discovery to show that the pharmacologic inhibition of TNIK has an anti-EMT effect in cancer cells; this effort could improve the druggability of TNIK, particularly regarding target validation. Therefore, in this study, we evaluated the effect of a novel aminothiazole inhibitor of TNIK, 5-(4-methylbenzamido)-2-(phenylamino)thiazole-4-carboxamide (hereinafter referred to as our database code number, KY-05009; Fig. 1*A*), on TGF-β-mediated EMT in human lung adenocarcinoma A549 cells [23]. The kinase selectivity assay revealed that KY-05009 also inhibited Mixed lineage kinase 1 (MLK1) as well as TNIK [23], but we here focused on its cellular activity relevant to TNIK.

## Materials and Methods

### Molecular Docking

All computational calculations for molecular docking were performed using the Schrödinger suite [24]. The Nck-interacting kinase structure (PDB ID 2X7F) [25] was revised in Protein Preparation Wizard, and KY-05009 was generated in LigPrep. The docking study was carried out with a grid box of 30×30×30 Å^3^, centered on the corresponding ligand using the Standard Precision (SP) protocol in Glide. The backbone carbonyl group of Glu106, the backbone nitrogen, and the carbonyl groups of Cys108 were selected as H-bond constraints.

### KY-05009 synthesis and determination of its binding constant (*K*_i_) for TNIK

KY-05009 was synthesized as described previously, with modifications (Figure S1) [23]. The inhibition activity of ATP binding to TNIK by KY-05009 was determined using an orthogonal ATP competition assay (*K*_d_ELECT, KINOME*scan*, DiscoveRx, USA).

### Cell Culture

A549 human lung epithelial adenocarcinoma cell line was purchased from ATCC (USA) and maintained in Dulbecco's modified Eagle's medium (DMEM; Hyclone, UT), containing 10% heat-inactivated fetal bovine serum (FBS) and 1% antibiotics (100 U/mL penicillin and 100 µg/mL streptomycin), in a humidified atmosphere of 5% CO_2_ at 37°C.

### Cell viability assay

A549 cells (5×10^3^ cells/well) were seeded in a 96-well plate, and incubated for 24 h. After serum starvation for 24 h, cells were treated with 5 ng/mL human recombinant TGF-β1 (R&D systems) and KY-05009 for 48 h. Cell viability was measured using the Cell Counting Kit-8 (Dojindo Molecular Technologies, Japan), according to the manufacturer's instructions. Absorbance was measured using the Wallac EnVision microplate reader (PerkinElmer, Finland). All experiments were performed in triplicate.

### TOP/FOPflash and NF-κB reporter luciferase assays

A549 cells were trasfected using TOPflash TCF reporter plasmid (wild type TCF binding site, Millipore), FOPflash plasmid (mutant TCF binding site, Millipore) and Lipofectamine^2000^ (Invitrogen) in an antibiotics-free medium. FOPflash-normalized TOPflash luciferase activity was represented to the relative TCF/LEF luciferase activity. A549 cells were also transfected using the NF-κB reporter (5×10^5^ TU; SABioscience), in an antibiotic-free medium. After 48 h, the medium was changed to culture medium containing 10% FBS, and transduced cells were selected in culture medium with 30 µg/mL of puromycin (Sigma, MO). After puromycin selection, cells (1×10^4^ cells/well) were incubated in a 96-well plate for 24 h. After TOP/FOPflash or NF-κB reporter transfection, serum deprived cells were treated with TGF-β1 and KY-05009 for 48 h. Cells were washed with PBS, then lysed using passive lysis buffer (Promega). Luciferase activity was evaluated using the luciferase reporter assay (Promega). All experiments were performed in triplicate.

### Western blot analysis

Cytoplasmic or nuclear protein fractions of A549 cell lysates were prepared using the RIPA buffer (Cell Signaling Technology, Inc.) or the NucBuster Protein Extraction kit (Novagen, Germany). After protein quantification, cytoplasmic or nuclear protein (40 µg) was loaded on 8–15% polyacrylamide gels, and separated proteins were transferred to PVDF membranes. Proteins were detected using specific primary antibodies against: TNIK, TCF4, β-catenin, vimentin, Twist, actin, and histone H3, purchased from Santa Cruz Biotechnology, Inc. (TX, USA); phospho (p)-ERK1/2, ERK1/2, p-Smad2 (S465/467), Smad2/3, Snail, p-FAK (Y925), FAK, p-Src (Y416), Src, p-paxillin (Y118), paxillin, p-JNK (Y183/Y185), and JNK, purchased from Cell Signaling Technology, Inc.; p-Ser, α-smooth muscle actin (α-SMA), and IgG, purchased from Abcam; and E-cadherin and N-cadherin, purchased from BD Biosciences and Millipore. After incubation with HRP-conjugated secondary antibodies, membranes were developed using SuperSignal West Femto Maximum Sensitivity Substrate (Pierce) and the LAS-3000 luminescent image analyzer (Fuji Photo Film Co., Ltd., Japan). Each analysis was repeated three times in order to check the reproducibility of result and ImageJ (NIH, USA) software-based quantification of the detected band was carried out in the images represented in each figure. The relative, normalized ratio between phosphorylated protein and the protein itself or the loading control was presented in each image of figures.

### Immunoprecipitation

A549 cells were seeded at a density of 1×10^5^ cells/mL, and cultured for 24 h. After serum starvation for 24 h, cells were treated with TGF-β1 and KY-05009 for 48 h. After protein quantification, total lysates were pre-cleared with normal control IgG and PureProteome™ Protein A magnetic beads (Millipore). Immunoprecipitation of endogenous protein complexes was carried out overnight at 4°C with an anti-TCF4 antibody in combination with 30 µL of PureProteome™ Protein A magnetic beads. TCF4-bound proteins were subjected to Western blot analysis.

### Immunocytochemistry

A549 cells (1×10^5^ cells/mL) were incubated on a 1% gelatin-coated slide glass for 24 h. After serum starvation for 24 h, cells were treated with TGF-β1 and KY-05009 for 48 h. All cells were fixed with 2% formaldehyde, permeabilized with 0.1% Triton X-100 in PBS, and blocked with 3% BSA in PBS. Expression of p-Smad2, E-cadherin, and vimentin was detected using each respective primary antibody, and visualized with Alexa Fluor 488-conjugated secondary antibodies (Invitrogen). Nuclei were counterstained with Hoechst 33258. All images were observed by DeltaVision RT wide-field epifluorescence microscope imaging system and softWoRxs image analysis program (Applied Precision, NW, USA).

### Migration assay

A549 cells (1.5×10^4^ cells/well) were incubated for 24 h in a bottom line-marked, 96-well ImageLock Microplate (ESSEN BioScience). After serum starvation for 24 h, wounds were made using a 96-well WoundMaker™ (ESSEN BioScience), and cells were treated with TGF-β1 and KY-05009. The continuous kinetic output of migrated cells was analyzed by IncuCyte™ software, every 12 h. The relative wound density (RWD) was calculated in comparison with a similar cell population that was treated with TGF-β1 alone. All experiments were performed in triplicate.

### Invasion assay

A549 cells (1×10^5^ cells/well) were incubated in a 24-well plate for 24 h. After serum starvation for 24 h, cells were treated with TGF-β1 and KY-05009 for 48 h. Cells were collected using trypsin-EDTA, and resuspended in serum-free medium for counting. Culture medium (30 µL) containing 10% FBS was added to the bottom of a Boyden chamber. After placing over the gelatin-coated membrane filter, the silicone gasket, and the top chamber, the cell suspension (2×10^4^ cells/50 µL) was added to the top chamber, followed by incubation at 37°C in 5% CO_2_ for 6 h. The membrane filter was collected, fixed, and stained using the Diff-Quick staining kit (Dade Behring), according to the manufacturer's instructions. After the filter was dried and stabilized on a glass slide using 30% glycerol solution, the migrated cells were counted in three randomly selected fields at 400× magnification. All experiments were performed in triplicate.

### Quantitative Real Time-PCR

A549 cells (1×10^5^ cells/mL) were incubated in a 6-well plate for 24 h. After serum starvation for 24 h, cells were treated with TGF-β1 and KY-05009 for 72 h. Total RNA was isolated using the TRIzol reagent (Life Technologies), and cDNA was synthesized using the Omniscript Reverse Transcriptase Kit (Qiagen), according to the manufacturer's instructions. Quantitative reverse transcriptase (RT)-PCR was performed using Brilliant SYBR Green Master Mix (Stratagene) and the Mx3000P Real-Time PCR system (Stratagene). Primer sequences used in this study were designed as the following: MMP-2, forward, 5′-TTG ACG GTA AGG ACG GAC TC-3′, reverse, 5′-ACT TGC AGT ACT CCC CAT CG-3′; MMP-9, forward, 5′- TTG ACA GCG ACA AGA AGT GG-3′, reverse, 5′-GCC ATT CAC GTC GTC CTT AT-3′; and GAPDH, forward, 5′-GAG TCA ACG GAT TTG GTC GT-3′, reverse, 5′-GATCTCGCTCCTGGAAGATG-3′. All reactions were run in triplicate, and data were analyzed using the 2^−ΔΔC^_T_ method [26]. GAPDH was used as an internal standard. Statistical significances were determined using the Student's *t*-test with GAPDH-normalized 2^−ΔΔC^_T_ values.

### Gelatin zymography

To analyze MMP-2 and MMP-9 activity, A549 cells (1×10^5^ cells/well) were incubated in a 24-well plate for 24 h. After serum starvation for 24 h, cells were treated with TGF-β1 (5 ng/mL) and KY-050095 for 48 h. The supernatants were collected, centrifuged at 3,000×*g* for 10 min, concentrated using Amicon Ultra Centrifugal Filter Units (Millipore), and quantified using the BCA protein assay kit (Pierce). Proteins (40 µg) were loaded onto a gelatin-containing acrylamide gel (8% acrylamide; 1.5 mg/mL gelatin), and separated by electrophoresis. Next, the gel was washed with 2.5% Tween-20 solution, developed overnight at 37°C in Zymogram incubation buffer (50 mM Tris-HCl, pH 7.6; and 5 mM CaCl_2_), stained with 0.25% Coomassie blue R250 solution, and destained with a solution of 50% methanol and 10% acetic acid until the part of membrane degraded by MMP-2 or MMP-9 became clear.

### Statistical analysis

Data are presented as mean ± SD. Statistical significance of independent three experiments was determined using the Student's *t*-test, and differences were considered significant, as follows: ^##^ *p*<0.01, ^###^ *p*<0.001 (versus the control); * *p*<0.05, ** *p*<0.01, *** *p*<0.001 (versus the cell population treated with TGF-β1 alone).

## Results

### Molecular docking and binding mode of KY-05009 to TNIK

Before evaluating the effect of KY-05009 on TGF-β-mediated EMT in human lung adenocarcinoma A549 cells, we investigated the binding mode and main interactions of KY-05009 with TNIK. As shown in Fig. 1B, there are two hydrogren bonds between KY-05009 and the backbone of Cys108 in the hinge region that play crucial roles in the mechanism by which KY-05009 inhibits TNIK. Leu160 and Val170 undergo CH/π interactions with the thiazole and benzamide of KY-05009 at a distance of 3.34 Å and 3.99 Å, respectively. Additionally, located between Val31 and Gly111, the benzene ring of KY-05009 stabilizes the binding mode with CH/π interactions.

### Inhibitory binding constant (*K*_i_) of KY-05009 for TNIK

The inhibitory binding constant, *K*_i_, of KY-05009 for TNIK was calculated from triplicate, 11-point dose-response curves, as shown in Fig. 1C. KY-05009 inhibited the binding of ATP to TNIK in a dose-dependent manner (*K*_i_ = 100 nM).

### KY-05009 inhibits TGF-β1-induced activation of Wnt signaling

**Figure 2:**
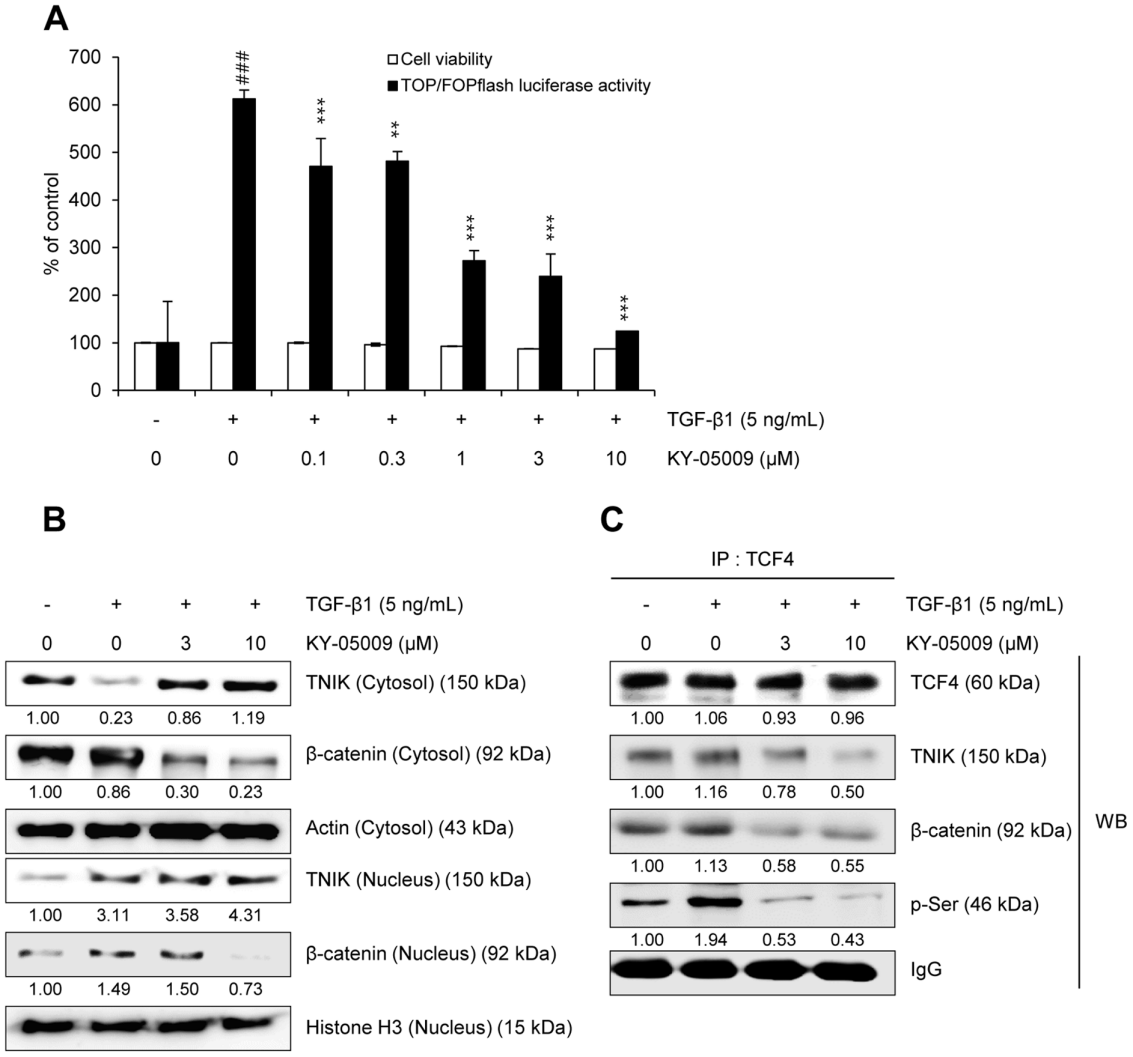
KY-05009 inhibits TGF-β1-induced Wnt signaling. (A) Effects of KY-05009 on cell viability and TCF4 transcriptional activity. Serum-deprived A549 cells were treated with TGF-β1 or its combination with KY-05009 for 48 h, and then cell viability and TOPflash luciferase activity were measured. FOPflash-normalized TOPflash luciferase activity was represented to the relative TCF/LEF luciferase activity. The expression of TNIK and β-catenin in cytosolic and nuclear fractions (B), and the protein levels of TCF4-interacting proteins c were measured by Western blot analysis and immunoprecipitation assay, respectively. Actin, histone H3, and IgG were used as loading controls. The expression of cytosol and nucleus proteins was normalized by actin and histone H3, respectively. Reported results are representatives of triplicate experiments. ^###^ *p*<0.001 (versus ‘the control’); ** *p*<0.01, *** *p*<0.001 (versus ‘the group treated with TGF-β1 only’).

To confirm the inhibitory effect of KY-05009 on TCF4-mediated transcription, and determine the concentration of KY-05009 used in this study, we conducted cell viability and TOP/FOPflash luciferase activity assays in parallel. As shown in Fig. 2A, KY-05009 did not cause significant cytotoxicity up to 10 µM, but it significantly inhibited the TGF-β1-mediated induction of TCF4-mediated transcription at 1–10 µM. Therefore, a concentration range of 3–10 µM was used for KY-05009 in the following experiments.

In the process of EMT, TGF-β1-induced activation of TCF4-mediated transcription is dependent on the protein level of β-catenin, and/or its nuclear translocation. As shown in Fig. 2B, TGF-β1 induced the nuclear translocation of β-catenin in A549 cells, while 10 µM KY-05009 completely inhibited this induction. KY-05009 also reduced β-catenin protein levels at 3–10 µM.

The binding of β-catenin to TCF4, and subsequent phosphorylation of TCF4, is necessary for TNIK to activate Wnt signaling [20], [21]. Here, TGF-β1 induced the phosphorylation of TCF4, and this induction was strongly inhibited by KY-05009 (Fig. 2C). Although KY-05009 did not alter the TGF-β1-induced increase in nuclear TNIK protein levels (Fig. 2B), it inhibited TNIK binding to TCF4 (Fig. 2C).

### KY-05009 inhibits TGF-β1-induced activation of Smad signaling

**Figure 3:**
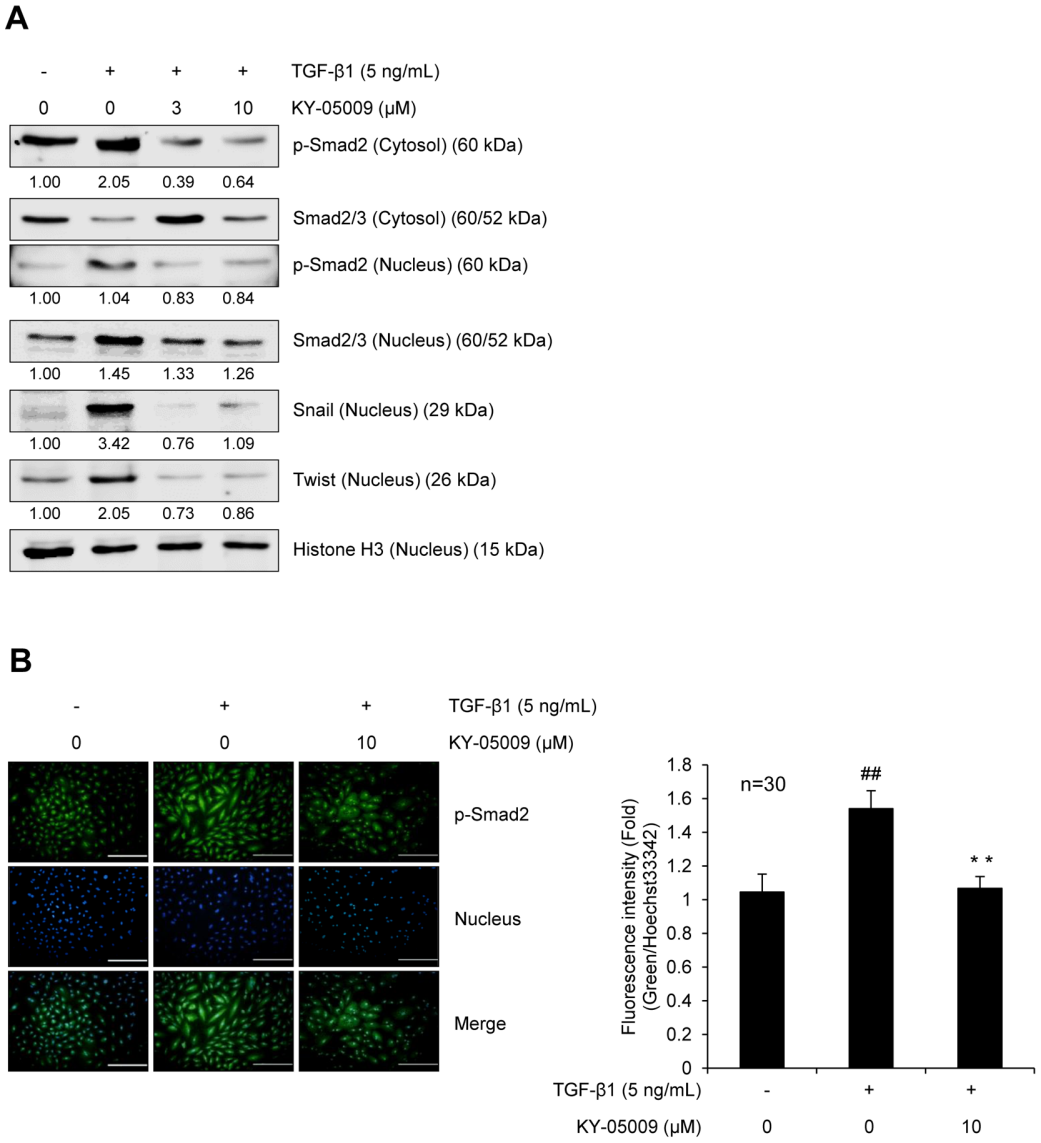
KY-05009 inhibits TGF-β1-induced Smad signaling. (A) Effects of KY-05009 on TGF-β1-activated Smad signaling. Serum-deprived A549 cells were treated with TGF-β1 or its combination with KY-05009 for 48 h, and then the levels of p-Smad2 and endogenous Smad2/3 in cytosolic and nuclear fractions were evaluated by Western blot analysis. The nuclear expression levels of Snail and Twist were also evaluated. The expression of cytosol p-Smad2 was normalized by endogenous Smad2/3 and all nucleus proteins were normalized by histone H3. (B) Immunocytochemical confirmation of KY-05009 inhibition of TGF-β1-activated p-Smad2. Nuclei were counterstained with Hoechst 33342. All scale bars represent 50 µm. The expression of p-Smad2 was represented by the relative intensity of green fluorescence (n = 30), and reported images are representatives of triplicate experiments. ^##^ *p*<0.01 (versus ‘the control’), ** *p*<0.01 (versus ‘the group treated with TGF-β1 only’).

To investigate the effect of KY-05009 on TGF-β1-induced activation of Smad signaling, protein levels of p-Smad2, Smad2/3, and the transcription factors, Snail and Twist, were evaluated. As shown in Fig. 3A, TGF-β1 strongly induced the phosphorylation of Smad2 and nuclear translocation of p-Smad and Smad2/3, while KY-05009 inhibited these inductions. The inhibitory effect of KY-05009 on TGF-β1-induced phosphorylation of Smad2 was also confirmed by immunocytochemistry analysis (Fig. 3B). Additionally, TGF-β1 strongly induced the protein expression of Snail and Twist, and KY-05009 also dramatically inhibited these inductions.

### KY-05009 inhibits TGF-β1-mediated modulation of EMT markers

**Figure 4:**
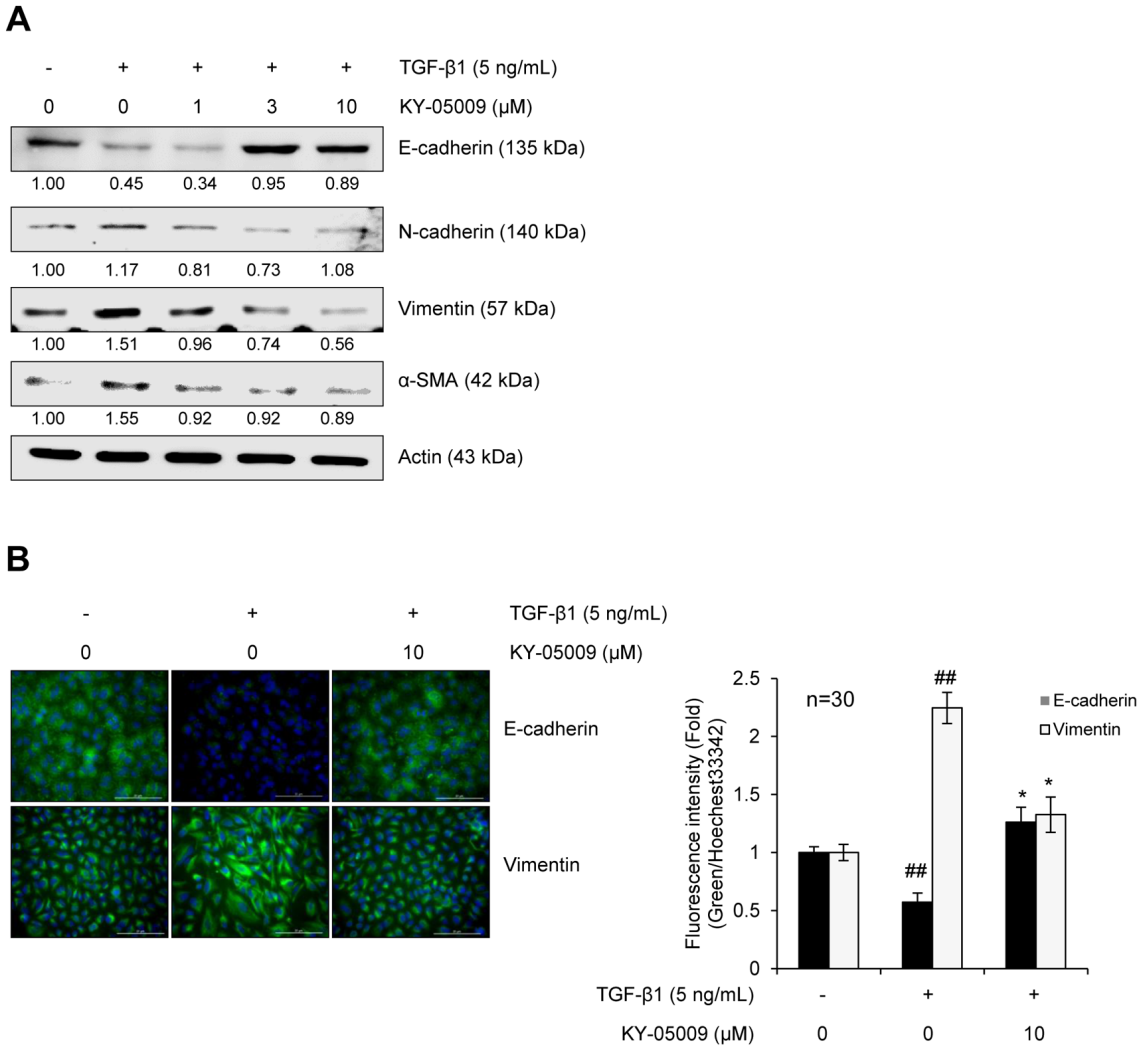
KY-05009 inhibits TGF-β1-mediated modulation of EMT markers. The effect of KY-05009 on TGF-β1-mediated modulation of EMT markers was evaluated by (A) Western blot analysis and (B) immunofluorescence microscopy. Serum-deprived A549 cells were treated with TGF-β1 or its combination with KY-05009 for 48 h. Actin was used as a loading control. Nuclei were counterstained with Hoechst 33342, and all scale bars represent 20 µm. The expressions of E-cadherin and vimentin were represented by the relative intensity of green fluorescence (n = 30), and reported images are representatives of triplicate experiments. ^##^ *p*<0.01,* *p*<0.05 (versus ‘the group treated with TGF-β1 only’).

To verify EMT inhibition by KY-05009, the effect of KY-05009 on the expression of epithelial and mesenchymal markers was evaluated. As shown in Fig. 4A, TGF-β1 reduced the expression of the epithelial marker, E-cadherin, but increased the expression of mesenchymal markers, N-cadherin and vimentin. KY-05009 treatment blocked these effects of TGF-β1. This inhibitory effect of KY-05009 on the TGF-β1-mediated change of EMT markers was also confirmed by immunocytochemistry analysis (Fig. 4B); both the TGF-β1-mediated decrease of E-cadherin and increase of vimentin were strongly attenuated by KY-05009.

### KY-05009 inhibits TGF-β1-induced migration and invasion

**Figure 5:**
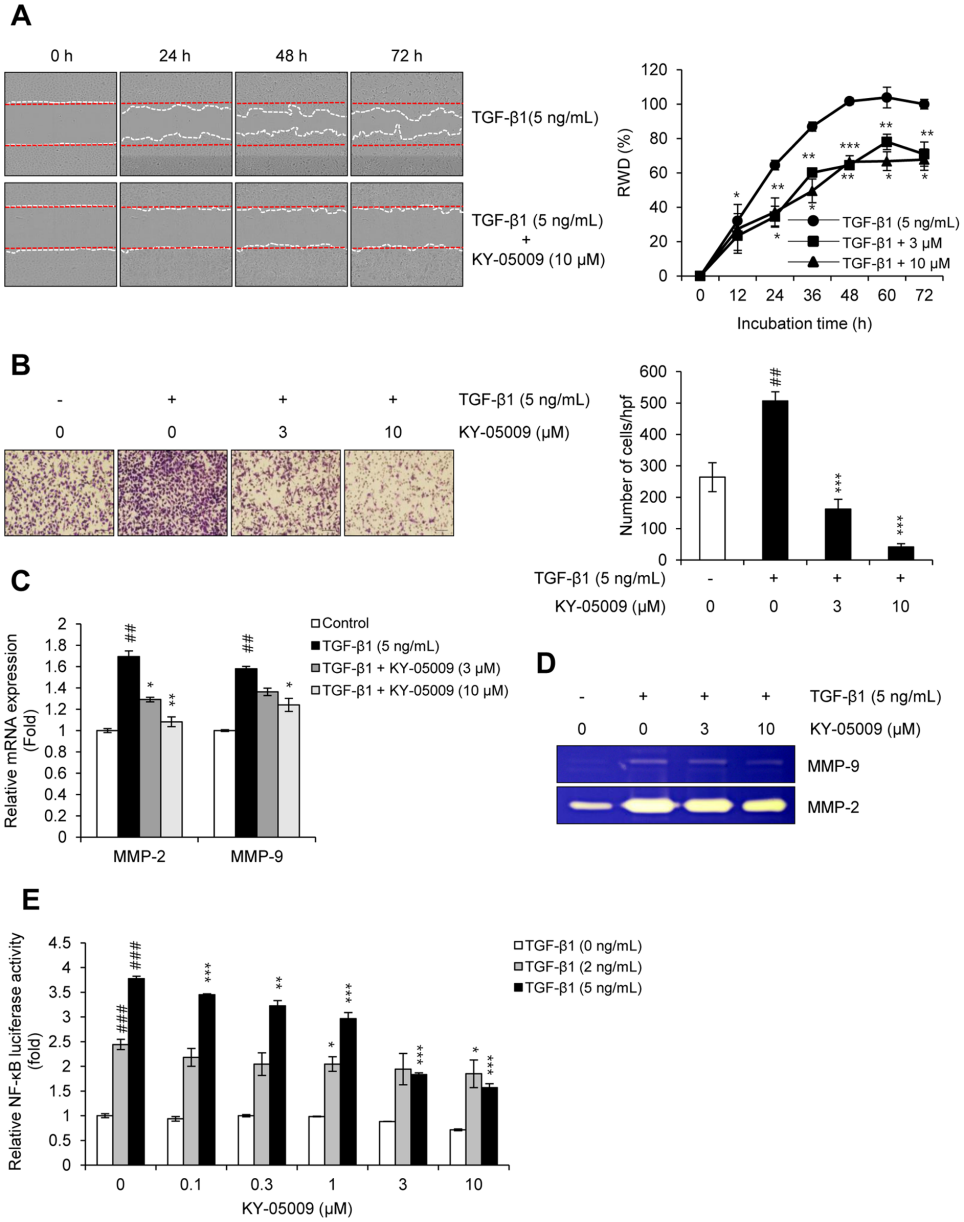
KY-05009 inhibits TGF-β1-induced migration and invasion. The effect of KY-05009 on TGF-β1-induced migration and invasion of A549 cells was evaluated using (A) IncuCyte software and (B) Boyden chambers, respectively. The red and white dashed lines a represent the wounded area and the edge of migrated cells, respectively. Values (% RWD; Relative Wound Density) represent mean ± SD of triplicate samples, and reported images are representatives of triplicate experiments. Numbers of invaded cells were represented by an average number of cells per randomly selected three high-power field (HPF). Effects of KY-05009 on TGF-β1-induced expression and activation of MMP-2 and MMP-9 were measured by (C) quantitative RT-PCR and (D) gelatin zymography, respectively. (E) The effect of KY-05009 on NF-κB transcriptional activity was determined by reporter assay. ^##^ *p*<0.01, ^###^ *p*<0.001 (versus ‘the control’); * *p*<0.05, ** *p*<0.01, *** *p*<0.001 (versus ‘the group treated with TGF-β1 only’).

Next, the effects of KY-05009 on TGF-β1-induced migration and invasion were investigated in A549 cells. As shown in Fig. 5A, the TGF-β1-induced migration of A549 cells was significantly inhibited by KY-05009. KY-05009 also significantly inhibited TGF-β1-induced invasion of A549 cells across the gelatin-coated membrane (Fig. 5B). Quantitative RT-PCR and gelatin zymography were used to further investigate the effect of KY-05009 on the TGF-β1-induced activation of the gelatinases, matrix metalloproteinase (MMP)-2 and MMP-9. TGF-β1 induced the mRNA expression levels and gelatinase activities of both MMP-2 and MMP-9, while KY-05009 inhibited these inductions (Fig. 5C and 5D). Furthermore, TGF-β1 induced the transcriptional activity of NF-κB, which is a transcription factor that regulates MMP-2 and MMP-9 expression, and is well-known to play a role cancer metastasis; KY-05009 also attenuated this effect in a dose-dependent manner (Fig. 5E).

### KY-05009 inhibits TGF-β1-induced activation of focal adhesion and other non-Smad signaling pathways

**Figure 6:**
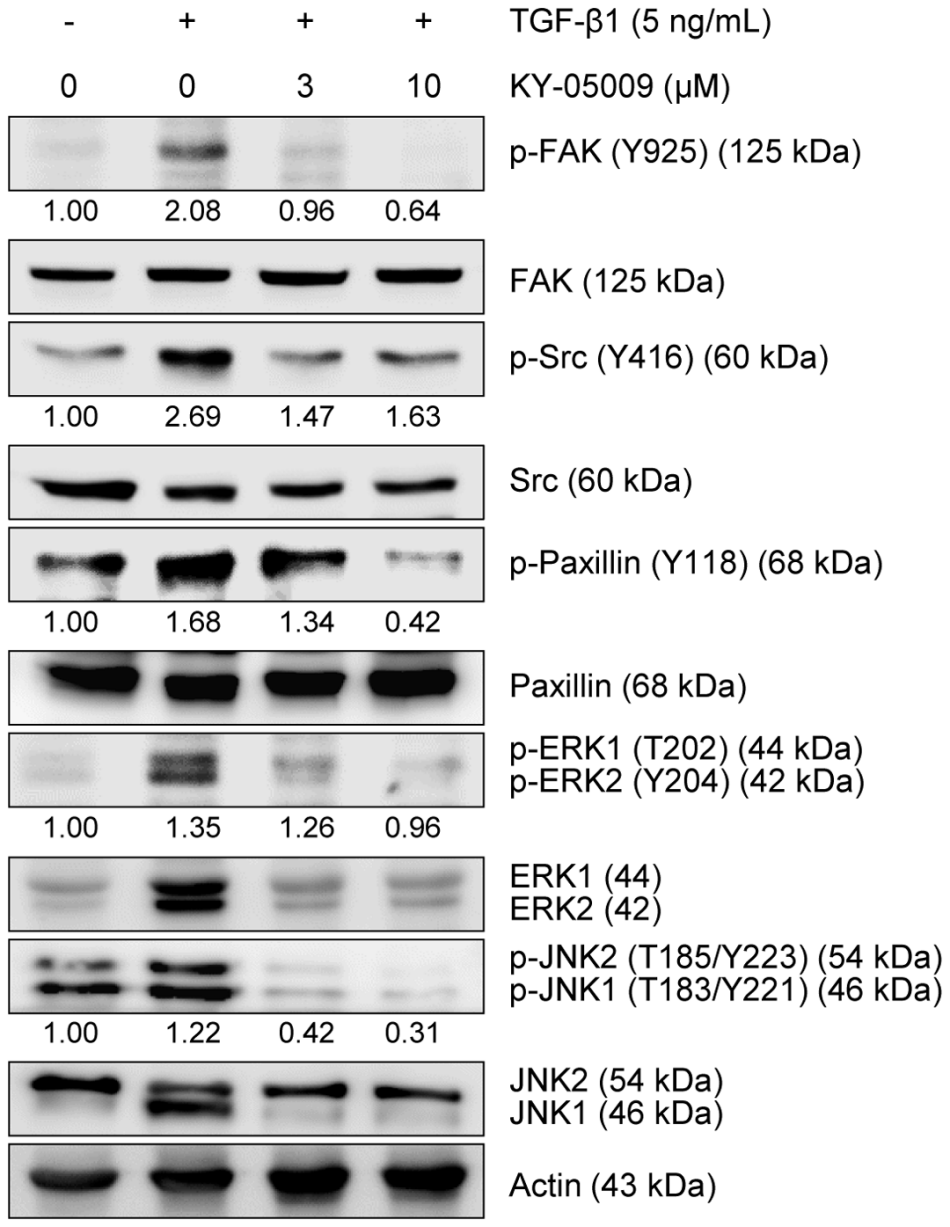
KY-05009 inhibits TGF-β1-induced activation of focal adhesion and non-Smad signaling pathways. Serum-deprived A549 cells were treated with TGF-β1 or its combination with KY-05009 for 48 h. Effects of KY-05009 on TGF-β1-induced activation of focal adhesion-related and ERK and JNK MAP kinase signaling molecules was evaluated by Western blot analysis. The ERK1 and 2 (p44 and p42, respectively) or JNK1 and 2 (p46 and p54, respectively) isoforms could be separated and differentially evaluated by gel electrophoresis, according to their molecular weight differences. The expression of p-FAK, p-Src, p-Paxillin, p-ERK1/2, and p-JNK2/1 was normalized by endogenous FAK, Src, Paxillin, ERK1/2, and JNK2/1, respectively. Actin was used as a loading control. Reported results are representatives of triplicate experiments.

Phosphorylation of focal adhesion kinase (FAK) at Y925 has been observed in different invasive tumors, and is associated with integrin adhesion dynamics and E-cadherin deregulation during EMT [27], [28]. Therefore, we further investigated the effect of KY-05009 on the activation of TGF-β1-induced focal adhesion and invasion-related signaling molecules, FAK, Src, and paxillin [29]. As shown in Fig. 6, TGF-β1 induced the phosphorylation of FAK (Y925), Src (Y416), and paxillin (Y118), and these phosphorylation events were inhibited by KY-05009.

In addition to its canonical, Smad-dependent pathway, TGF-β can also control EMT through non-Smad signaling pathways, including those of the MAP kinases, MEK/ERK and JNK [30], [31]. Therefore, the inhibitory effects of KY-05009 on TGF-β1-induced activation of ERKs and JNKs were investigated. KY-05009 inhibited the TGF-β1-induced phosphorylation of ERK1/2 and JNK1/2, as well as the TGF-β1-induced increase in JNK1 protein levels (Fig. 6).

## Discussion

TNIK was first identified in a yeast two-hybrid screen, and it was named for its interaction with both Traf2 and Nck [32]. It belongs to a subgroup of the Ste20 family of kinases, the germinal center kinase (GCK) family, which all have an N-terminal kinase domain and a C-terminal regulatory region. TNIK has been shown to regulate several signaling molecules and events, but the focus of this study was on its involvement in TGF-β1-induced EMT, as hypothesized from its ability to control both the Wnt and Smad signaling pathways, which are both important for EMT [20]–[22]. In order to validate TNIK as a druggable, anti-cancer target molecule, it is essential to verify the pharmacologic relevance of targeting TNIK. Thus, to address this relevance, we performed this study to investigate the effects of KY-05009 with the potential to inhibit TNIK activity, on TGF-β-induced EMT in A549 cells.

Recently, KY-05009 was shown to be a potent inhibitor of TNIK (IC_50_, 9 nM in a kinase assay), attenuating β-catenin/TCF4-mediated transcription [23]. It also inhibited MLK1 (IC_50_, 18 nM in a kinase assay), but we focused on its cellular activity relevant to TNIK. Reported here, the molecular docking/binding study revealed that KY-05009 has two H-bond interactions with Cys108 in the hinge region of TNIK, and CH/π interactions with Val31, Gly111, and Leu160. Furthermore, the inhibitory activity of KY-05009 against TNIK was confirmed by an ATP competition assay (*K*_i_, 100 nM).

KY-05009 inhibition of TGF-β1-induced activation of TCF4-mediated transcription in A549 cells is consistent with a previous study, which showed similar results in colorectal cancer cells [23]. One possible mechanism for this effect could be through KY-05009 inhibition of ATP binding to TNIK, which is downstream of TGF-β1 signaling, thus blocking the phosphorylation and activation of TCF4. In this study, TGF-β1 strongly induced the nuclear translocation of TNIK protein, but KY-05009 did not change this increased nuclear protein level. However, KY-05009 inhibited serine phosphorylation of TCF4, and attenuated the formation and activity of the TGF-β1-induced TNIK-TCF4-β-catenin complex, which culminates in canonical Wnt signaling. These results provide evidence that KY-05009 inhibits TGF-β1-mediated Wnt signaling through its inhibition of the kinase activity of TNIK, which phosphorylates TCF4. In further support, TNIK has been shown to interact with TCF4 through amino acids 1–289, which includes the kinase domain [21]. KY-05009 also caused a decrease in β-catenin protein levels, providing another possible mechanism through which it inhibits TGF-β1-induced activation of Wnt signaling.

In its canonical pathway, TGF-β1 binding to its receptors leads to phosphorylation of Smad2/3, which then associates with Smad4, and translocates into the nucleus, where Smad complexes regulate specific target gene expression by interacting with transcriptional cofactors, such as β-catenin, Snail, and Twist. Increased expression of these cofactors can sensitize cells to TGF-β-induced EMT. Additionally, β-catenin-Smad2/3 complexes are rapidly formed during TGF-β-induced EMT [33], and Wnt signaling directly induces the expression of Snail [34]. Here, we showed that KY-05009 strongly inhibited TGF-β1-induced phosphorylation and nuclear translocation of Smad2 and the expression of Snail and Twist. KY-05009 also inhibited the TGF-β1-induced nuclear translocation of Smad2/3, but at the highest concentration tested (10 µM), it appeared to reduce the expression or stability of cytosolic Smad2/3. From these results, we hypothesized that the pharmacologic inhibition of TNIK by KY-05009 could inhibit TGF-β1-induced EMT through the attenuation of both Smad activation and expression of its transcriptional cofactors, such as β-catenin and Snail.

E-cadherin, N-cadherin, and vimentin have all been shown to be regulated by the Snail family and Twist transcription factors [35], [36], and β-catenin-Smad2/3 complexes have been reported to transcriptionally activate expression of α-SMA [33]. The translocation of β-catenin from the membrane into the nucleus has also been observed during the loss of E-cadherin [37], suggesting that EMT-related gene expression could be regulated by the complicated crosstalk between Wnt and Smad signaling pathways. Here, we showed that TGF-β1 caused a decrease in E-cadherin and increases in N-cadherin, vimentin, and α-SMA levels, and KY-05009 blocked these effects. Our results suggest that KY-05009 blocks these effects through its inhibitory actions on both the Wnt and Smad signaling pathways.

Through loss of epithelial and acquisition of mesenchymal characteristics, tumor cells undergoing EMT become more motile and invasive, allowing for metastatic spread. Here, we showed that TGF-β1 strongly induced the migration and invasion of A549 cells, and KY-05009 significantly inhibited these inductions. One possible mechanism through which KY-05009 inhibits invasion could be through its attenuation of TGF-β1-induced expression and activation of MMP-2 and MMP-9 via NF-κB, which is a well-known transcription factor that controls cancer metastasis [38].

The functionally interdependent protein kinases (i.e., FAK and Src) and focal adhesion molecules (i.e., paxillin) are also involved in TGF-β1-induced EMT [39]–[41]. Hyperactivation of FAK has been reported in cancer cell lines and cancer metastasis [42], and Src-mediated regulation of E-cadherin and vimentin has also been reported in several cancers [43], [44]. Paxillin is an oncogene that is involved in cell migration; it is highly expressed in lung cancer tissues, and positively correlates with increased EMT [45]. Additionally, enhanced paxillin phosphorylation has been observed in breast cancer cells derived from mouse lung metastases [46]. Here, we showed that TGF-β1 strongly induced the phosphorylation of FAK, Src, and paxillin, while KY-05009 inhibited these inductions.

Activation of MAP kinases is also involved in TGF-β1-induced EMT. The activation of ERK is required for TGF-β1-induced EMT *in vitro* [47]. Consistent with a previous study [48], TGF-β1 strongly induced phosphorylation of ERK1/2, while KY-05009 completely inhibited this induction. Several studies have shown that TGF-β1 can induce JNK phosphorylation, and constitutively active JNK can cause cancer cell migration and invasion, and induces the expression of mesenchymal-specific markers, vimentin and fibronectin [31], [49]. JNK signaling has also been shown to regulate cancer cell migration through phosphorylation of paxillin [50]. JNK1 is also able to promote TGF-β1-induced EMT through its interaction with Smad3 [51], and its down-regulation blunts TGF-β1-mediated EMT in epithelial cells [52]. Furthermore, inhibiting JNK1 activation attenuates TGF-β1-mediated EMT-related modulation of E-cadherin and α-SMA expression [53]. Interestingly, TNIK can promote the activity of JNK2, and activation of both JNK1 and JNK2 can be blocked by TNIK knockdown, suggesting that TNIK is required for the activation of the JNK signaling pathway [32], [54], [55]. In this study, TGF-β1 strongly induced the expression of JNK1 and the phosphorylation of JNK1/2 in A549 cells, while KY-05009 completely inhibited these effects.

Cross-talk between several signaling pathways plays a role in the acquisition of EMT in cancer cells. Therefore, targeting molecule(s), which are involved in the process of EMT, might be sufficient to control cancer metastasis. Although the possible involvement of MLK1 on the anti-EMT activity of KY-05009 could not be excluded, we suggested in this study that the pharmacologic inhibition of TNIK by KY-05009 could suppress TGF-β-activated EMT in A549 cells through the attenuation of downstream Smad and non-Smad signaling pathways, including the Wnt, NF-κB, FAK-Src-paxillin-related focal adhesion, and MAP kinase (ERK and JNK) pathways. These findings imply that pharmacologic inhibition of TNIK is a possible new method to control EMT, which contributes to metastatic processes in cancers. The next step is to identify more potent inhibitors of TNIK, and to perform functional studies to solidify the druggability of TNIK as an anti-EMT target, using siRNA and/or expression of a dominant negative form. This study substantiates the continuous efforts to identify and validate potential therapeutic targets associated with EMT, such as TNIK, that may provide new and improved therapies for treating and/or preventing EMT-based disorders, such as cancer metastasis and fibrosis.

## Supporting Information

Figure S1 **Synthesis of KY-05009.** **Step 1: Preparation of ethyl 2-cyano-2-(hydroxyimino)acetate.** Acetic acid (6.9 g, 115 mmol) was added to a suspension of ethyl cyanoacetate (10 g, 88 mmol) and sodium nitrite (7.3 g, 106 mmol) in water (40 mL) at 0–5°C over a period of 1 h. The temperature was slowly raised to room temperature, and the reaction mixture was stirred for 1 h at that temperature. After the complete consumption of ethyl cyanoacetate (monitored by TLC), the reaction mixture was extracted with ethyl acetate (5×150 mL). The combined organic layer was successively washed with 10% sodium bicarbonate (2×150 mL) and brine solution (125 mL), and dried over sodium sulfate. The solvent was removed under reduced pressure. The resulting solid was stirred with *n*-hexane (300 mL) for 30 minutes at room temperature, then filtered and dried under vacuum to afford 11 g (88% yield) of title compound. ^1^H-NMR (300 MHz, CDCl_3_) δ (ppm) 9.02 (br, 1H), 4.46 (q, 2H, J = 7.1 Hz), 1.42 (t, 3H, 7.4 Hz). **Step 2: Preparation of ethyl 2-amino-2-cyanoacetate.** The H-cube system was charged with a Pt/C CatCart column and was heated to 25°C. The hydrogen pressure was set to 10 bars. Ethyl 2-cyano-2-(hydroxyimino)acetate (7.3 g, 52 mmol) was dissolved in EtOH (250 mL), and the solution was pumped through the H-Cube system with a flow rate of 1 mL/min for 2 days. The collected solution was concentrated under reduced pressure to produce a yellowish oil. The solution was evaporated under reduced pressure to produce 6.4 g (96% yield) of the title compound as a pale yellowish oil. ^1^H-NMR (300 MHz, CDCl_3_) δ (ppm) 4.43 (s, 1H), 4.34 (q, 2H, J = 7.1 Hz), 1.95 (br, 2H), 1.36 (t, 3H, 7.2 Hz). **Step 3: Preparation of ethyl 5-amino-2-(phenylamino)thiazole-4-carboxylate.** Phenyl isothiocyanate (1.5 g, 9.0 mmol) was added to a suspension of ethyl 2-amino-2-cyanoacetate (1.0 g, 8.2 mmol) in THF (17 mL), and the mixture was refluxed for 3 h. The solvent was evaporated, and the residue was purified by silica gel column chromatography, eluted with 5% MeOH in DCM to produce 1.2 g (52% yield) of the titled compound. ^1^H-NMR (300 MHz, CDCl_3_) δ (ppm) 9.56 (s, 1H), 7.50 (d, 2H, J = 8.1 Hz), 7.28–7.23 (m, 1H), 6.70–6.85 (m, 3H), 4.21 (t, 2H, J = 7.1 Hz), 1.27 (t, 3H, J = 7.02 Hz). **Step 4: Preparation of ethyl 5-(4-methylbenzamido)-2-(phenylamino)thiazole-4-carboxylate.** *p*-toluoyl chloride (0.39 g, 2.5 mmol) was added to a solution of ethyl 5-amino-2-(phenylamino)thiazole-4-carboxylate (0.66 g, 2.5 mmol) in pyridine (12 mL), and the mixture was heated at 60°C for 12 h. The solvent was evaporated, and the residue was purified by silica gel column chromatography, eluted with 5% MeOH in DCM to produce 0.4 g (56% yield) of the titled compound. ^1^H-NMR (300 MHz, CDCl_3_) δ (ppm) 11.58 (s, 1H), 7.89 (d, 2H, J = 8.4 Hz), 7.40-7.30 (m, 5H), 7.18 (s, 1H), 7-13-7.08 (m, 1H), 4.49 (q, 2H, J = 7.1 Hz), 1.47 (t, 3H, J = 7.3 Hz). **Step 5: Preparation of 5-(4-methylbenzamido)-2-(phenylamino)thiazole-4-carboxamide.** 7M NH_3_ in MeOH (3 mL) was added to a solution of ethyl 5-(4-methylbenzamido)-2-(phenylamino)thiazole-4-carboxylate (0.13 g, 0.33 mmol) in THF (1 mL), and the solution was heated in a sealed tube at 80°C for 12 h. The solvent evaporated, and the resulting solid was collected. The solids were washed with ether and dried to produce 50 mg (43% yield) of the title compound. ^1^H-NMR (300 MHz, CDCl_3_) δ (ppm) 12.09 (s, 1H), 7.89 (d, 2H, J = 8.5 Hz), 7.41-7.36 (m, 3H), 7.33-7.30 (m, 2H), 7.09 (t, 1H, J = 6.9 Hz), 6.90 (m, 1H), 6.80 (s, 1H), 5.49 (s, 2H), 2.44 (s, 3H).
(TIF)
