# Supplementary figures and images for: A Novel Aminothiazole KY-05009 with Potential to Inhibit Traf2- and Nck-Interacting Kinase (TNIK) Attenuates TGF-β1-Mediated Epithelial-to-Mesenchymal Transition in Human Lung Adenocarcinoma A549 Cells
Source: PLoS One. 2014 Oct 22;9(10):e110180. doi: 10.1371/journal.pone.0110180 (PMC4206343; doi:10.1371/journal.pone.0110180)

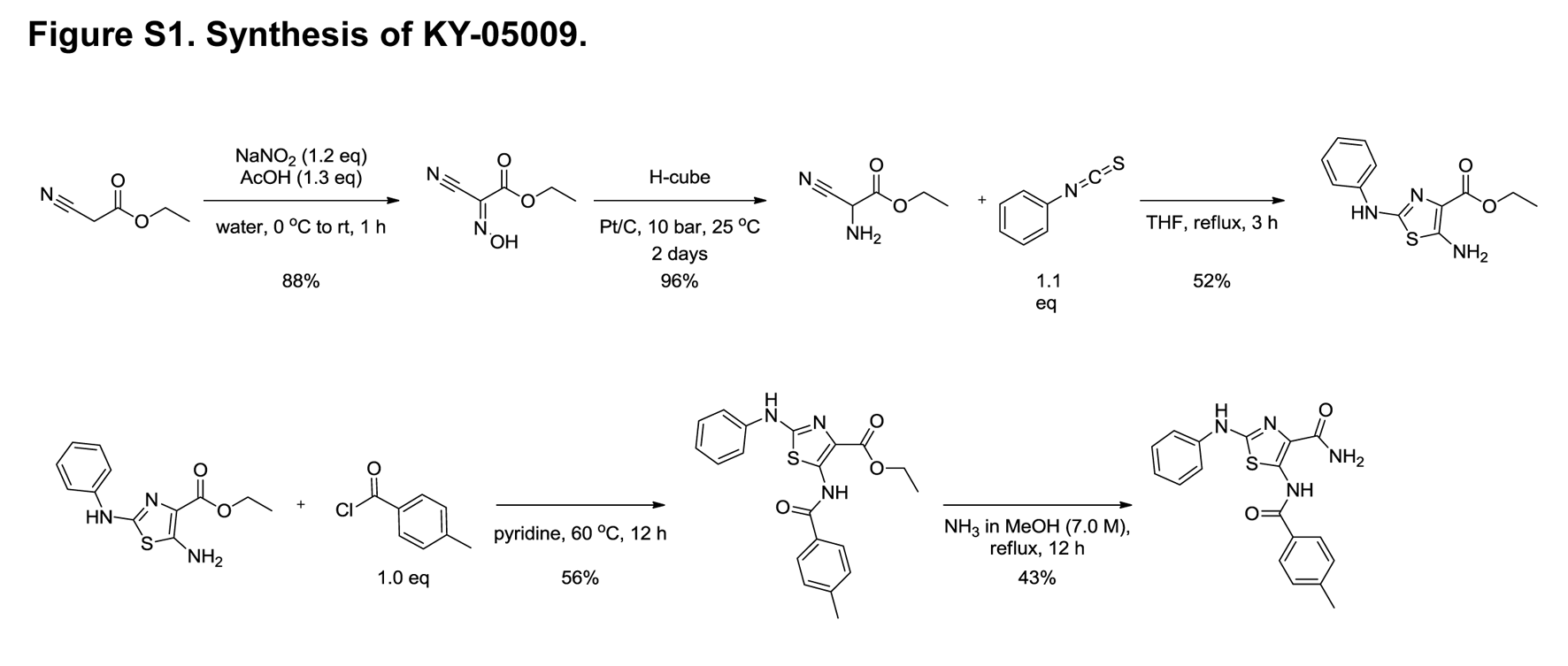

Supplement: Figure S1 — Synthesis of KY-05009. Step 1: Preparation of ethyl 2-cyano-2-(hydroxyimino)acetate. Acetic acid (6.9 g, 115 mmol) was added to a suspension of ethyl cyanoacetate (10 g, 88 mmol) and sodium nitrite (7.3 g, 106 mmol) in water (40 mL) at 0–5°C over a period of 1 h. The temperature was slowly raised to room temperature, and the reaction mixture was stirred for 1 h at that temperature. After the complete consumption of ethyl cyanoacetate (monitored by TLC), the reaction mixture was extracted with ethyl acetate (5×150 mL). The combined organic layer was successively washed with 10% sodium bicarbonate (2×150 mL) and brine solution (125 mL), and dried over sodium sulfate. The solvent was removed under reduced pressure. The resulting solid was stirred with n-hexane (300 mL) for 30 minutes at room temperature, then filtered and dried under vacuum to afford 11 g (88% yield) of title compound. 1H-NMR (300 MHz, CDCl3) δ (ppm) 9.02 (br, 1H), 4.46 (q, 2H, J = 7.1 Hz), 1.42 (t, 3H, 7.4 Hz). Step 2: Preparation of ethyl 2-amino-2-cyanoacetate. The H-cube system was charged with a Pt/C CatCart column and was heated to 25°C. The hydrogen pressure was set to 10 bars. Ethyl 2-cyano-2-(hydroxyimino)acetate (7.3 g, 52 mmol) was dissolved in EtOH (250 mL), and the solution was pumped through the H-Cube system with a flow rate of 1 mL/min for 2 days. The collected solution was concentrated under reduced pressure to produce a yellowish oil. The solution was evaporated under reduced pressure to produce 6.4 g (96% yield) of the title compound as a pale yellowish oil. 1H-NMR (300 MHz, CDCl3) δ (ppm) 4.43 (s, 1H), 4.34 (q, 2H, J = 7.1 Hz), 1.95 (br, 2H), 1.36 (t, 3H, 7.2 Hz). Step 3: Preparation of ethyl 5-amino-2-(phenylamino)thiazole-4-carboxylate. Phenyl isothiocyanate (1.5 g, 9.0 mmol) was added to a suspension of ethyl 2-amino-2-cyanoacetate (1.0 g, 8.2 mmol) in THF (17 mL), and the mixture was refluxed for 3 h. The solvent was evaporated, and the residue was purified by silica gel column [file pone.0110180.s001.tif]
